# Waardenburg-Shah syndrome (WS type IV): a rare case from Pakistan

**DOI:** 10.1186/s13741-019-0135-x

**Published:** 2020-01-24

**Authors:** Taimoor Ashraf Khan, C. Aqeel Safdar, Shehryar Zameer, Arshad Khushdil

**Affiliations:** National University of Medical Sciences, Rawalpindi, Pakistan

**Keywords:** Waardenburg-Shah Syndrome, Waardenburg syndrome type IV, Hirschsprung’s disease, Total intestinal aganglionosis

## Abstract

Waardenburg-Shah syndrome is a rare autosomal recessive [AR] inherited disorder characterized by the presence of Hirschsprung’s disease with a high likelihood of aganglionic megacolon, due to which the mortality is high. The management of the condition involves surgical intervention for the removal of the aganglionic segment of the colon. Here, we report a neonate that presented with a white forelock, white eyelashes, iris hypopigmentation, and sensorineural deafness associated with bilious vomiting, refusal to feed, and failure to pass meconium indicating intestinal obstruction.

## Introduction

Waardenburg syndrome (WS) is a rare inherited disorder of autosomal recessive type with variable clinical presentation. On the basis of cluster of genotypic and phenotypic presentations, there are four different types of this syndrome ranging from type I to type IV (or Waardenburg-Shah syndrome). Types III and IV are the least common types (Mahmoudi et al. 2013). The classic presentations of Waardenburg-Shah syndrome (WS type IV) include sensorineural deafness, hypopigmentation of the hair, the skin, irises, and Hirschsprung disease (HD) (Shah et al. 1981).

To the best of our knowledge, there are < 80 cases of Waardenburg syndrome type IV (WS type IV) described in the scientific literature, with an incidence of approximately 1/40,000–1/50,000 live births (Cui et al. 2013). Here, we report a case of Waardenburg syndrome type IV from Pakistan that presented on natal day with a clinical picture of intestinal obstruction and aganglionosis. This is the first case of Waardenburg syndrome type IV with total intestinal aganglionosis from Pakistan.

## Case report

A boy on his natal day was received in Neonatal Intensive Care Unit (NICU) of Pak Emirates Military Hospital (PEMH) Rawalpindi, Pakistan, on the 22nd of May 2017 with complaints of bilious vomiting, refusal to feed, and failure to pass meconium. He was delivered through spontaneous vaginal delivery at full term but had delayed cry according to attending nurse. His mother had gestational hypertension and his parents had consanguineous marriage. Two other siblings were alive and healthy.

On examination, he was lethargic but otherwise vitally stable, had a white forelock, white eyelashes, blue colored irises, and no response to loud noise. He had good Moro but poor sucking reflex. The patient was admitted to NICU and was managed for vomiting which settled after 3 h. His abdomen started distending with palpable gut loops on the 2nd post-natal day (PND). He also developed persistent vomiting and jaundice. The patient had empty rectum on digital rectal examination (DRE) with no gush of air or stools. His stomach was decompressed by nasogastric intubation and kept nil per oral. X-ray erect and supine, USG (ultrasonography), and CT (computed tomography) scan abdomen were done and surgical review was sought. Phototherapy was started for jaundice which subsided after 2 days. On the 3rd post-natal day, exploratory laparotomy was done and dilated small gut up to the ileum, collapsed distal ileum about 2 ft proximal from ileocecal junction, and thread-like colon were found. Ileostomy was done and biopsies from ileostomy stoma site, appendix, transverse colon, and sigmoid were sent for histopathology. Ileostomy stoma site biopsy revealed lack of ganglion cells, suggesting total colon Hirschsprung’s disease. Patient was managed conservatively by that time as stoma was fully functional till the 8th post-op day but then the patient gradually developed abdominal distension by the 10th post-op day. The site of ileostomy started oozing and became non-functional on the 11th post-op day. Thus, re-do ileostomy was performed on the same day. Small amount of stools were passed through the stoma, but the abdominal distension did not relieve and increased gradually. Patient’s condition was critical on the 21st post-natal day, developed acidotic breathing with persistent abdominal distension, and absent bowel sounds. He had no nasogastric aspirate and no fecal output from the stoma. The 2nd exploratory laparotomy was performed, and multiple full thickness biopsies were taken for frozen section which showed ganglion cells in the distal jejunum. The distal jejunum was then brought out as loop stoma and previous stoma was closed. Abdominal distension was relieved on the 2nd post-op day and bowel sounds became positive. He had a fully functional jejunostomy stoma and was managed in the NICU for the next 1 week. Ophthalmological and ENT were sought confirming heterochromiairidia and sensorineural deafness. Genetic studies were not performed. The patient improved gradually and was discharged home on the 28th post-natal day. The patient was followed up in OPD for next 2 months and was doing well on outdoor treatment but then he was lost to follow-up.

## Discussion

This report highlights an extremely rare incident case of Waardenburg syndrome type IV in Pakistan along with the inevitable role of frozen section in the correct management of the condition. This patient presented with iris hypopigmentation and poliosis as white forelock, white eyelashes associated with bilious vomiting, deafness, refusal to feed, and failure to pass meconium indicating intestinal obstruction.

Scarcely, Waardenburg syndrome type IV might present with mild neuronal symptoms, especially when the SOX10 (SRY-related HMG-box gene 10) gene is involved (Wildhardt et al. 2013). Waardenburg proposed the diagnostic criteria for Waardenburg syndrome types, later modified by Krishnakumar N. Shah (for type IV) which includes sensorineural hearing loss and hypopigmentation along with Hirschsprung disease. Hirschsprung disease is characterized by agangliosis of the intestines, caused by the failed migration of colonic ganglion cells during gestation. There is absence of the sub-mucous plexus and the myenteric plexus due to which fecal matter cannot pass, resulting in obstruction of the colon which is a characteristic of Waardenburg syndrome type IV (Shim et al. 1999; Bansai et al. 2013).

Mutations of different genes like MITF (microphthalmia-associated transcription factor), PAX3 (Paired Box Gene 3), SOX10, and EDNRB (endothelin receptor type B, gene) have been noted in causing mild or incomplete phenotypic expression of symptoms in Waardenburg syndrome (Wildhardt et al. 2013). Mutations in EDNRB gene that encodes for the endothelin-B receptor are the culprits in humans. Within the Pakistani population, there is a high likelihood that mutations of the EDNRB gene are the causative mutation of Waardenburg syndrome type IV (Jabeen et al. 2012).

In our case, both parents as well as the two siblings did not have clinical features of Waardenburg’s syndrome. The parents were also first cousins. Although not conclusive, the available genetic information in this case suggests that an autosomal recessive inheritance is most likely.

For treatment of intestinal agangliosis, surgical intervention is required. Appropriate management demands early clinical diagnosis supported by investigations and per-operative frozen sections. Audiological assessment at birth and at periodic intervals can detect hearing impairment. Parents must be provided with genetic counseling for the next pregnancy.

## Conclusion

Shah-Waardenburg syndrome is a very rare syndrome but has a very high morbidity and mortality in the neonatal age group due to total intestinal aganglionosis. These patients have debilitating outcomes if timely management is not done. Pre-operative frozen sections have pivotal role in appropriate management of such patients. Like other autosomal recessive diseases, consanguineous marriages are responsible for increasing incidence in Pakistan. Poor health care facilities, lack of awareness, and illiteracy add to the plight of these children. Better outcomes warrant a multidisciplinary approach to these patients.

## Data Availability

Data sharing is not applicable to this article as no datasets were generated or analyzed.

## Abbreviations

AR: Autosomal recessive
CT: Computed tomography
DRE: Digital rectal examination
HD: Hirschsprung’s disease
NICU: Neonatal Intensive Care Unit
PND: Post-natal day
POD: Post-operative day
USG: Ultrasonography
WS: Waardenburg-Shah
